# Hepatitis C Virus Infection Increases Risk of Gallstone Disease in Elderly Chinese Patients with Chronic Liver Disease

**DOI:** 10.1038/s41598-018-22896-4

**Published:** 2018-03-15

**Authors:** Xu Li, Pujun Gao

**Affiliations:** Department of Hepatology, The First Hospital of Jilin University, Jilin University, No. 71 Xinmin Street, Changchun, 130021 China

## Abstract

We investigated possible links between the etiology of liver disease and gallstone risk in Chinese patients with chronic liver disease (CLD). We compared the outcomes of 267 Chinese CLD patients with gallstones and those of a control group of 1,015 CLD patients without gallstones. Logistic regression analyses adjusting for demographic features and other gallstone risk factors revealed that liver cirrhosis increased the risk of gallstone development twofold [adjusted odds ratio (AOR); 95% confidence interval (95% CI): 2.343 (1.710–3.211)]. HCV infection increased gallstone risk 1–2-fold [AOR; 95% CI: 1.582 (1.066–2.347)] higher than did HBV infection. Multivariate analyses of the risk of developing gallstones in patients with liver cirrhosis after an HCV or HBV infection yielded an estimated AOR (95% CI) of 1.601 (1.063–2.413) in patients with an HCV infection. In elderly patients with CLD (≥60 years of age), gallstone risk also increased significantly after an HCV infection [AOR (95% CI): 2.394 (1.066–5.375)]. HCV infection, older age, and liver cirrhosis significantly correlate with an increased risk of gallstone development in Chinese patients with CLD. HCV infection further increases this risk in both patients with liver cirrhosis and in elderly CLD patients (≥60 years of age).

## Introduction

Gallstone disease (GD) is a common and costly digestive disorder^1–3^ that has been diagnosed in 10–20% of adults in the developed world. The main risk factors for gallstone formation are gender (females have a higher risk), advanced age, obesity, alcohol use, diabetes, and hypertriglyceridemia^4,5^. In China, the number of individuals with chronic liver disease (CLD) is significant; its major known causes include infection with hepatitis B virus (HBV) or hepatitis C virus (HCV) and excessive alcohol consumption.

Determining the potential relationship between the development of gallstones and the etiology of CLD is of great interest, as previous research has suggested that HCV infection is a relevant risk factor for gallstone formation^6^. Recent studies have found that gallstones may occur more frequently in patients infected with HCV than in either patients infected with HBV or alcoholics^7–10^. In Italy, a multicenter survey demonstrated that GD prevalence among patients with liver cirrhosis was associated with HCV infection but not with excessive alcohol consumption^11^. However, few studies have been conducted on the relationship between the etiologies of CLD and GD in elderly adults^6^.

Thus, the current study was undertaken to specifically analyze the risk factors for the development of gallstones in Chinese CLD patients, as well as the possible relationships between HCV infection and gallstone formation in subsets of CLD patients, such as those with liver cirrhosis and the elderly.

## Results

### Demographic and clinical characteristics of study participants

Baseline demographic and clinical characteristics of the study participants (N = 1282) are presented in Table 1. The case group was composed of 267 CLD patients with gallstones, including 114 male and 153 female patients with a median age of 61.00 years. In addition, 186 patients (69.7%) had cirrhosis, 220 patients (82.4%) had chronic HCV (CHC), and 44 patients (16.5%) had diabetes. The control group consisted of 1,015 CLD patients without gallstones. The median age of the control group was 56.00 years, and approximately one-half (43.6%) were male. Within this group, 525 patients (51.7%) had cirrhosis, 794 patients (78.2%) had CHC, and 151 patients (14.9%) had diabetes.

**Table 1:** Demographic and clinical characteristics of study participants.

Variable	No gallstones n = 1015	Gallstones n = 267	*P*
Male, n (%)	443 (43.6)	114 (42.7)	0.781
Age (years)	56.00 (49.00,63.00)	61.00 (54.00,68.00)	<0.001
AST (IU/L)	55.00 (33.70,91.00)	56.00 (38.20, 99.00)	0.077
ALT (IU/L)	51.00 (29.00,97.93)	48.50 (27.90,98.00)	0.335
TBIL (µmol/L)	20.70 (13.50,34.60)	22.40 (15.80,44.80)	0.004
ALB (g/L)	35.80 (30.40,39.60)	33.20 (28.50,38.00)	<0.001
Diabetes, n (%)	151 (14.9)	44 (16.5)	0.516
Liver disease etiology			0.136
HBV, n (%)	221 (21.8)	47 (17.6)	
HCV, n (%)	794 (78.2)	220 (82.4)	
Liver cirrhosis, n (%)	525 (51.7)	186 (69.7)	<0.001

Continuous variables are expressed as the median (25th and 75th percentiles). Categorical variables are displayed as numbers and percentages.

AST: aspartate aminotransferase, ALT: alanine aminotransferase, TBIL: total bilirubin, ALB: albumin, HBV: hepatitis B virus, HCV: hepatitis C virus.

We observed no significant differences in clinical characteristics, including levels of aspartate aminotransferase (AST) and alanine aminotransferase (ALT), between the two groups. In the case group, total bilirubin (TBIL) levels were significantly higher than in the control group. However, in the case group, albumin (ALB) levels were significantly lower than in the control group.

### Univariate and multivariate analyses of variables associated with gallstones in CLD patients

Results of the univariate analysis suggested that the distributions of age and liver cirrhosis significantly differed between the case and control groups. Gender, age, levels of TBIL, diabetes, liver cirrhosis, and liver disease etiology were thus considered for multivariable analysis. The adjusted odds ratio (AOR) for patients with liver cirrhosis was 2.343 [95% confidence interval (CI): 1.710–3.211; *P* < 0.001] compared to patients without cirrhosis (Table 2). The AOR for patients with HCV-related CLD was 1.582 (95% CI: 1.066–2.347; *P* = 0.023) compared to patients with HBV-related CLD. In addition, the AOR for older patients (≥60 years of age) was 1.848 (95% CI: 1.378–2.477; *P* < 0.001) compared to younger patients (<60 years of age). However, we found no significant association between TBIL levels and gallstone formation.

**Table 2:** Univariate and multivariate analyses of variables associated with gallstones in CLD patients.

Variable	Non-gallstones n = 1015	Gallstones n = 267	*P* ^*#*^	AOR (95% CI)*	*P* ^**^
Gender			0.781	—	—
Female, n (%)	572 (56.4)	153 (57.3)			
Male, n (%)	443 (43.6)	114 (42.7)			
Age			<0.001	1.848 (1.378–2.477)	<0.001
<60 years, n (%)	658 (64.8)	122 (45.7)			
≥60 years, n (%)	357 (35.2)	145 (54.3)			
TBIL			0.103	—	—
<30 µmol/L, n (%)	703 (69.3)	171 (64.0)			
≥30 µmol/L, n (%)	312 (30.7)	96 (36.0)			
Diabetes			0.516	—	—
No, n (%)	864 (85.1)	223 (83.5)			
Yes, n (%)	151 (14.9)	44 (16.5)			
Liver disease etiology			0.136	1.582 (1.066–2.347)	0.023
HBV, n (%)	221 (21.8)	47 (17.6)			
HCV, n (%)	794 (78.2)	220 (82.4)			
Liver cirrhosis			<0.001	2.343 (1.710–3.211)	<0.001
No, n (%)	490 (48.3)	81 (30.3)			
Yes, n (%)	525 (51.7)	186 (69.7)			

AOR: adjusted odds ration; CI: confidence interval.

^#^*P* value for univariate analysis.

*Adjusted for gender, age, TBIL level, diabetes, etiology, and liver cirrhosis.

^**^P value for multivariate analysis.

TBIL: total bilirubin, HBV: hepatitis B virus, HCV: hepatitis C virus.

### Liver cirrhosis, age, and gallstone development

Because liver cirrhosis is believed to be a major risk factor for gallstone development in CLD patients, we evaluated the association of risk factors and gallstone formation in 711 patients with cirrhosis (Table 3).

**Table 3:** Association of gallstone development with cirrhosis of different viral etiologies.

Variables	No gallstones n = 525	Gallstones n = 186	*P* ^#^	AOR (95% CI)*	*P* ^**^
Gender			0.803	—	—
Female, n (%)	330 (62.9)	115 (61.8)			
Male, n (%)	195 (37.1)	71 (38.2)			
Age			<0.001	1.712 (1.181–2.481)	0.005
<60, n (%)	322 (61.3)	81 (43.5)			
≥60, n (%)	203 (38.7)	105 (56.5)			
TBIL			0.680	—	—
<30, n (%)	290 (55.2)	106 (57.0)			
≥30, n (%)	235 (44.8)	80 (43.0)			
Diabetes			0.324	—	—
No, n (%)	440 (83.8)	150 (80.6)			
Yes, n (%)	85 (16.2)	36 (19.4)			
Liver disease etiology			<0.001	1.601 (1.063–2.413)	0.024
HBV, n (%)	213 (40.6)	47 (25.3)			
HCV, n (%)	312 (59.4)	139 (74.7)			
Child class			0.509	—	—
A, n (%)	200 (38.1)	62 (33.3)			
B, n (%)	222 (42.3)	84 (45.2)			
C, n (%)	103 (19.6)	40 (21.5)			

Continuous variables are expressed as the median (25th and 75th percentiles).

^#^*P* value for univariate analysis.

*Adjusted for gender, age, TBIL level, diabetes, etiology, and Child class.

^**^P value for multivariate analysis.

TBIL: total bilirubin, HBV: hepatitis B virus, HCV: hepatitis C virus.

Results of the univariate analysis suggested that the distributions of etiology and age significantly differed between patients with and without gallstones. Gender, age, TBIL levels, diabetes, severity of cirrhosis, and etiology were included in the multivariable analysis. The AOR for patients with HCV-related CLD was 1.601 (95% CI: 1.063–2.413; *P* = 0.024) compared to patients with HBV-related CLD. In addition, the AOR for older patients was 1.712 (95% CI: 1.181–2.481; *P* = 0.005) compared to younger patients. However, we found no significant association between the severity of cirrhosis and gallstone formation between patients with cirrhosis, regardless of age.

We further analyzed the relationship between gallstone risk and gender, TBIL levels, diabetes, etiology, and liver cirrhosis in CLD patients of different ages (Table 4). In elderly CLD patients, the AOR for patients with HCV-related CLD was 2.394 (95% CI: 1.066–5.375; *P* = 0.034) compared to patients with HBV-related CLD. The AOR for patients with liver cirrhosis was 2.193 (95% CI: 1.430–3.362; *P* < 0.001) compared to patients without liver cirrhosis. In younger patients with CLD, the AOR for patients with liver cirrhosis was 2.062 (95% CI: 1.374–3.093; *P* < 0.001) compared to patients without cirrhosis. However, we did not find any significant association between liver disease etiology and gallstone formation in CLD patients younger than 60 years of age.

**Table 4:** Association of gallstone development and viral etiology in CLD patients of different ages.

Variable	Age ≥ 60 years		Age < 60 years	
	AOR (95%CI)	**P	AOR (95% CI)	**P
Liver cirrhosis		<0.001		<0.001
No, n (%)	1		1	
Yes, n (%)	2.193 (1.430–3.362)		2.062 (1.374–3.093)	
Liver disease etiology		0.034		—
HBV, n (%)	1			
HCV, n (%)	2.394 (1.066–5.375)			

*Adjusted for gender, TBIL, diabetes, etiology, and liver cirrhosis.

**P value for multivariate analysis.

CLD: chronic liver disease, HBV: hepatitis B virus, HCV: hepatitis C virus.

## Discussion

In the present study, patients with CHC infection had a higher prevalence of GD than did chronic HBV (CHB)-infected patients. This finding is in agreement with that of previous studies^11,12^. Stroffolini *et al*.^11^ assessed GD prevalence among patients with liver disease and the association between GD and the severity and etiology of hepatic injury; they found that subjects with HCV-related cirrhosis (OR 2.13, 95% CI: 1.38–3.26) had a higher risk of GD than subjects with HBV-related cirrhosis. Wijarnpreecha *et al*.^12^ conducted a meta-analysis to assess the risk of gallstone development in HCV-infected patients; these authors found that such patients had a significantly increased risk of developing gallstones compared to those without an HCV infection.

Several mechanisms could explain the greater risk of GD in HCV-infected patients^8^. First, both gallbladder dysfunction and altered bile composition affect gallstone formation in CHC patients. Chronic hemolysis secondary to hypersplenism, hyperestrogenism, changes in biliary lipid proportions, low hepatic synthesis and transport of bile salts, and unconjugated bilirubin all contribute to gallstone development in CLD patients. Some studies also found that a viral infection of the gallbladder may increase gallstone formation by altering gallbladder mucosal function or dysmotility^13–15^; further investigations are needed to address this hypothesis.

Second, direct HCV infection of the gallbladder may play an important role in gallstone development. Damage to the bile duct is a histological characteristic of chronic HCV infection, which has been found to be a risk factor for intrahepatic cholangiocarcinoma^16^. The HCV core protein could also promote the malignant transformation of human biliary epithelial cells^17^. Loriot *et al*.^18^ found that the concentration of HCV RNA was the same in serum, bile, and cultures of gallbladder epithelial cells, and levels of HCV particles isolated from gallbladder epithelial cells support this statement. HCV RNA and antigens have been detected in gallbladder autopsy specimens from HCV-infected patients^19^.

Third, insulin resistance has been shown to increase in both cholesterol GD^20–22^ and CHC infection in patients with visceral obesity, regardless of the genotype of HCV or severity of liver damage^23–25^. Increases in insulin resistance may provide a link between CHC infection and GD, perhaps via increases in the number of bile-saturated cholesterol molecules^8^. In addition, the association between the HCV nonstructural protein NS5A and both lipid droplets and apoA1 suggests that both NS5A and the core protein may play roles in the pathogenesis of aberrant lipid metabolism and contribute to liver steatosis, both commonly observed in patients with HCV infection^26^. The association between fatty liver disease and increased gallstone development has been linked to obesity and increased insulin resistance^27–30^.

Certain studies have analyzed the relationship between hepatitis viruses and GD in the elderly. Lee *et al*.^6^ conducted a study of the association between hepatitis and GD in elderly adults and found that both HBV and HCV were associated with GD in this population. In our study, we found that HCV infection led to a 2–3-fold greater number of gallstones than did HBV infection in elderly patients with CLD. However, there were no significant differences in GD occurrence in CLD patients younger than 60 years of age. As mentioned above, chronic liver inflammation, HCV infection of gallbladder epithelial cells^31^, and certain metabolic factors are known to be involved in GD development in CHC patients. Prior studies have also suggested that HBV is capable of increasing GD frequency via the infection of gallbladder epithelial cells^32^ and chronic liver inflammation. Our results suggest that it is possible that metabolic factors, including altered lipid and glucose metabolism, might strongly induce gallstone formation in elderly patients.

We also discovered that patients with liver cirrhosis have a twofold higher number of gallstones than patients without cirrhosis, which is in line with results from previous studies^11,33–36^. This finding could be explained by changes in bile composition and impaired gallbladder motility in patients with cirrhosis^15,37,38^. However, we did not find any correlation between the extent of gallstone formation and the severity of cirrhosis. One reason for this finding might be due to the inconsistent causes of liver cirrhosis in our study. An inconsistent etiology could influence the extent of liver cirrhosis, changes in bile composition, or impairments in gallbladder motility^6^, which may lead to GD. Moreover, we did not find a positive correlation between TBIL levels and gallstone formation. Correlations between TBIL levels and GD remain controversial and needed to be further discussed combined with gallstone type^7,39^.

Our study has several limitations. It had a retrospective design with little detail of HCV RNA and HBV DNA levels, or of other investigations of the associations between disease etiology, HCV RNA, HBV DNA, and GD development. The case and control patients were specifically selected from those seeking medical care at our hospital, which permitted us to obtain sufficient patient samples. Our study did not examine GD type; thus, we could not discuss risk factors for different types of GD.

Because this study was retrospective, we could not acquire any information about blood lipids or other diseases such as hemolytic anemia, which affect the formation of GD. Additionally, we could not analyze the relationship between such diseases and GD development in CLD patients. Furthermore, despite including patients with a greater than 3-year history of CLD, we were unable to discern the causal relationship between HCV and the development of GD. Aprospective study with a larger sample size is needed to further analyze this causality.

In conclusion, we found that the risk of gallstone development in Chinese CLD patients was significantly associated with the occurrence of liver cirrhosis, older age, and HCV infection. Furthermore, patients infected with HCV formed more gallstones than did patients infected with HBV.

## Patients and Methods

### Patients

We conducted a retrospective, case-control study of patients from The First Hospital of Jilin University in China between January 2015 and November 2017. All methods were carried out in accordance with the approved guidelines.Patients with CHC and CHB infections were recruited for the study. A diagnosis of CHC infection was made if the patient had detectable levels of anti-HCV antibodies and serum HCV RNA for at least 6 months. Patients with CHB diagnosed by persistent or intermittent elevations in alanine transaminase level (≥twice the normal upper limit) and elevated levels of HBV DNA were observed for at least 6 months.

Potential participants were excluded if any of the following criteria were present: (i) co-infection with human immunodeficiency virus; (ii) history or evidence of a form of hepatitis other than that caused by HCV or HBV infection; or (iii) the presence of another liver disease, such as alcoholic liver disease; (iv) history of CLD less or equal than 3 years.

The Independent Institutional Review Board of The First Hospital of Jilin University approved the recruitment of human participants and our study protocol. Each participant provided written informed consent prior to enrollment in the study.

### Diagnosis of liver cirrhosis and GD

We confirmed each patient’s diagnosis of liver cirrhosis with either a liver biopsy or a combination of clinical, biochemical, and radiological findings. GD diagnosis was dependent on either the ultrasonographic detection of an echogenic structure within the gallbladder lumen that caused a posterior acoustic shadow^40^ or cholecystectomy findings.

### Diagnosis of diabetes

A diagnosis of diabetes was made in patients with either a known history of diabetes treated with an anti-diabetic therapy or at least one of the following criteria: (1) fasting glucose level greater than or equal to 7.0 mmol/L, (2) random glucose level greater than or equal to 11.1 mmol/L, and (3) 2-h postprandial plasma glucose level greater than or equal to 11.1 mmol/L^41^.

### Study variables

Demographic and clinical presentation variables in this study included gender, age, liver disease etiology, presence of diabetes, and cirrhosis. We also analyzed biochemical parameters, such as ALT, AST, TBIL, and ALB. In addition, the Child-Pugh score was calculated in patients with liver cirrhosis for the classification of cirrhosis severity. Abdominal ultrasound was also performed in cirrhosis patients to determine the extent of ascites and confirm the presence of cirrhosis.

### Statistical analysis

Continuous variable values are presented as the median (25th and 75th percentiles), whereas categorical variables are displayed as numbers and percentages; two-tailed, independent sample *t* tests and Chi-square analyses were employed to investigate continuous and categorical variables, respectively. Multivariate logistic regression analysis was used to adjust for possible confounding effects among the variables. We also calculated AORs and 95% CIs for these comparisons. We used SPSS software, version 13.0 (SPSS Inc., Chicago, IL, USA) for data analysis, and a *P* value less than 0.05 was considered statistically significant.
